# Sexual Dimorphism in Healthy Aging and Mild Cognitive Impairment: A DTI Study

**DOI:** 10.1371/journal.pone.0037021

**Published:** 2012-07-02

**Authors:** Laurence O’Dwyer, Franck Lamberton, Arun L. W. Bokde, Michael Ewers, Yetunde O. Faluyi, Colby Tanner, Bernard Mazoyer, Desmond O’Neill, Máiréad Bartley, Rónán Collins, Tara Coughlan, David Prvulovic, Harald Hampel

**Affiliations:** 1 Department of Psychiatry, Psychosomatic Medicine and Psychotherapy, Goethe University, Frankfurt, Germany; 2 Centre for Imaging-Neurosciences and Applications to Pathologies, UMR 6232, CNRS, CEA, University of Caen and Paris Descartes, Caen, France; 3 Cognitive Systems Group, Discipline of Psychiatry, School of Medicine and Trinity College Institute of Neuroscience (TCIN), Trinity College Dublin, Dublin, Ireland; 4 Department of Radiology, VA Medical Center, University of California San Francisco, San Francisco, California, United States of America; 5 Liaison Psychiatry Service, Addenbrooke’s Hospital, Cambridge, United Kingdom; 6 Cambridgeshire and Peterborough National Health Service (NHS) Foundation Trust, Cambridge, United Kingdom; 7 Department of Zoology, Trinity College Dublin, Dublin, Ireland; 8 Centre Hospitalier Universitaire, Caen, France; 9 Institut Universitaire de France, Paris, France; 10 Department of Medical Gerontology, Trinity College Dublin, Dublin, Ireland; Banner Alzheimer’s Institute, United States of America

## Abstract

Previous PET and MRI studies have indicated that the degree to which pathology translates into clinical symptoms is strongly dependent on sex with women more likely to express pathology as a diagnosis of AD, whereas men are more resistant to clinical symptoms in the face of the same degree of pathology. Here we use DTI to investigate the difference between male and female white matter tracts in healthy older participants (24 women, 16 men) and participants with mild cognitive impairment (21 women, 12 men). Differences between control and MCI participants were found in fractional anisotropy (FA), radial diffusion (DR), axial diffusion (DA) and mean diffusion (MD). A significant main effect of sex was also reported for FA, MD and DR indices, with male control and male MCI participants having significantly more microstructural damage than their female counterparts. There was no sex by diagnosis interaction. Male MCIs also had significantly less normalised grey matter (GM) volume than female MCIs. However, in terms of absolute brain volume, male controls had significantly more brain volume than female controls. Normalised GM and WM volumes were found to decrease significantly with age with no age by sex interaction. Overall, these data suggest that the same degree of cognitive impairment is associated with greater structural damage in men compared with women.

## Introduction

Many studies have found that the clinical symptoms of Alzheimer’s disease (AD) are not tightly linked to brain pathology [1]–[4]. This is underlined by post mortem findings which indicate that up to 25% of individuals who fulfil the neuropathological criteria for AD dementia remain cognitively normal during their lifetime [4]. The degree to which pathology translates into clinical symptoms has been shown to be strongly dependent on sex with women more likely to express pathology as a diagnosis of AD dementia, whereas men are more resistant to clinical symptoms in the face of the same degree of pathology [3]. This may be partly related to differences in brain structure, including the fact that the absolute brain volume of women is less than that of men [5], [6]. A higher synaptic density, greater number of neurons and larger brain size might partly explain why men appear to have a greater buffer against pathology translating into clinical symptoms of AD [7], [8]. Larger brain size, greater synaptic density, as well as education and a cognitively demanding occupation, have been suggested to contribute to an increased brain reserve that results in more efficient cognitive networks that can delay the onset of clinical symptoms of dementia [1], [9]–[13]. Some studies suggest that larger brains are less likely to develop AD [10] but this has not always been supported [14].

While AD usually entails significant memory loss, an intermediate state between healthy ageing and AD, termed mild cognitive impairment (MCI) has become widely recognized and a significant proportion of those with MCI may represent a prodromal state of AD [15]. White matter (WM) tracts may be key indicators of early pathology as these tracts are uniquely vulnerable to damage [16] and studies have shown WM damage both in MCI [17]–[20] and AD [21]–[24]. MRI studies have also indicated WM volume loss in AD dementia [25], although GM loss appears to be more pronounced [26], [27].

Diffusion tensor imaging (DTI) offers a powerful tool for the investigation of WM *in vivo* [28]–[30] but to our knowledge no DTI studies have yet looked at differences in white matter integrity between men and women in MCI or AD dementia, despite the fact that age and sex are the two highest risk factors for AD dementia [31]–[35].

In addition to the scarcity of DTI studies examining sex differences in the WM of MCI and AD patients, there are also very few studies which have examined WM differences between healthy older men and women. Studies in younger cohorts have shown that men have higher FA than women in the corpus callosum [36]–[38]. Men have also been shown to have higher FA and lower MD in the midcingulum bundles [39] while a TBSS study has shown lower FA values in the superior longitudinal fasciculus, forceps minor and corticospinal tract in normal adult women compared to men [40]. However another TBSS study found that women had higher FA in the fronto-occipital fasciculus, the body of the corpus callosum and parahippocampal WM, but lower FA in the bilateral internal capsule, frontal gyrus, fusiform gyrus, hippocampus, insula and postcentral gyrus [41]. All of the above studies involved cohorts ranging from ∼8 years to ∼40 years, and thus do not relate well to elderly cohorts.

The incidence of MCI and AD dementia in men and women has also been subject to much debate. For those greater than 80 years of age, increased incidence of AD dementia for women may be due to the greater longevity of women versus men. Nevertheless, even when correcting for age, women appear to be more affected by dementia than men [3], [32]–[35].

In elderly cohorts, sex differences in brain areas that are essential to higher cognitive functions have been found, with one study indicating that age-related cerebral volume loss and glucose metabolic deficit were significantly different between men and women [42]. PET studies have shown differences in the regional cerebral metabolic rate of glucose in AD dementia patients with men showing significantly greater hypometabolism compared with women with the same degree of cognitive impairment [1], [11].

In the current study, tract based spatial statistics (TBSS) was used to assess sex differences in healthy controls and MCI participants. TBSS obviates the need for *a priori* selection of regions of interest and provides a complete voxelwise analysis of the brain. Our hypothesis was that at an equivalent cognitive level, men would have greater WM microstructural damage compared with women This might suggest that men have a greater degree of brain reserve than women [13] as they are maintaining cognition at an equivalent level to their female counterparts despite the presence of greater WM damage. We also hypothesized that older women are at greater risk of developing MCI as they may meet the criteria for this condition despite having significantly less WM microstructural damage than men with a comparable level of MCI.

## Materials and Methods

### Ethics Statement

The study was approved by the St. James’ Hospital and Adelaide & Meath Hospital incorporating the National Children’s Hospital Research Ethics Committee and was in accordance with the Declaration of Helsinki. All participants provided informed written consent.

### Participants

Scans were obtained from the following groups of participants: 40 healthy older people, 33 MCI participants. The total number of participants was 73. MCI patients were diagnosed using criteria for both amnestic and non-amnestics sub-groups [43]. Neuropsychological assessment consisted of the Mini Mental State Examination (MMSE) [44] and the Consortium to Establish a Registry for Alzheimer’s Disease (CERAD) neuropsychological battery [45]. For the diagnosis of MCI, the following must be present:

1. objective impairment on any neuropsychological test from the CERAD battery based on a cut-off of −1.5 SD below published normative data corrected for age and education of the subject;
2. cognitive impairment corroborated by a close family member;
3. essentially normal activities of daily living;
4. must not meet criteria for dementia as defined below.

MCI individuals with objective memory impairment were diagnosed as having MCIa and those with non-memory impairment were diagnosed as having MCIna.

MCI participants were recruited at the Adelaide and Meath Hospital incorporating the National Children’s Hospital (AMNCH), Dublin, Ireland. Healthy participants were recruited among relatives of patients and also through advertisements in the local community.

Participants were excluded if they had cortical infarction, excessive subcortical vascular disease, space-occupying lesions, depression, and any other psychiatric or neurological disease. Participants were also excluded on magnetic resonance imaging criteria such as pacemaker implant, recent metallic implants, and claustrophobia. The DTI and structural scans of the cohort used in the current study were previously used in a study of mixed-effects models [19] and in a study of the role of multiple indices of diffusion in MCI and AD [18].

In the healthy older control group there was no significant difference between men and women in terms of age, education, and all of the psychological tests, except for Praxis Recall where men scored significantly better than women (Table 1).

**Table 1:** Demographic and Cognitive Characteristics of the Sample Groups.

	n		Age		Education		MMSE		Boston	
	Male	Female	Male	Female	Male	Female	Male	Female	Male	Female
Con	16	24	68.31 (8.41)	64.08 (6.81)	14.43 (6.91)	12.14 (2.00)	29.25 (1.44)	29.33 (0.82)	14.50 (0.73)	17.79 (0.41)
MCI	12	21	71.33 (6.97)	66.24 (6.46)	11.92 (3.48)	13.14 (3.00)	27.75 (3.02)	28.33 (3.34)	12.67 (1.87)	12.67 (1.68)
	**n**		**Word List Average**		**Word Recall**		**Praxis**		**Praxis Recall**	
	**Male**	**Female**	**Male**	**Female**	**Male**	**Female**	**Male**	**Female**	**Male**	**Female**
Con	16	24	7.50 (0.92)	7.39 (1.17)	8.19 (1.22)	8.50 (1.29)	10.75 (0.58)	10.54 (0.78)	**11.38 (1.78)**	**10.00 (1.87)**
MCI	12	21	**4.69 (1.37)**	**6.95 (1.60)**	**4.75 (2.14)**	**6.76 (2.21)**	9.75 (1.48)	9.62 (1.69)	8.67 (3.08)	9.43 (3.75)

Values are mean (SD). Abbreviations: Con, control; MCI, Mild cognitive Impairment; MMSE, Mini-Mentral State Examination. Significant differences between male and female groups are marked in bold where p<0.05 using a t-test.

For the MCI group there was no significant difference between men and women in terms of age, education, MMSE, or the Boston test. Female MCI participants scored significantly better than males MCI participants in Word List Average, Word Recall, Praxis and Praxis Recall (Table 1). Student’s t-tests were used to assess differences between groups with significance set at p<0.05. Statistical analyses were carried out with the R software package [46].

### Imaging Methods

Magnetic resonance imaging (MRI) was conducted with a Philips Achieva 3.0 Tesla MR system (Best, The Netherlands). A parallel SENSitivity Encoding (SENSE) approach [47] was used. The high resolution 3D T1-weighted structural images were achieved with the following pulse sequence: TR = 8.4 ms; TE  = 3.9 ms; flip angle  = 8°; number of axial slices  = 180; slice thickness  = 0.9 mm; acquisition voxel size  = 0.9×0.9×1.8 mm^3^; rec voxel size  = 0.9×0.9×0.9 mm^3^; field of view (FOV)  = 230 mm×230 mm×230 mm; acquisition matrix  = 256×256; SENSE reduction factor  = 2.3; total acquisition time  = 5 min 44 sec.

DTI was acquired using an echo planar imaging (EPI) sequence with the following pulse sequence: TR  = 12396 ms; TE  = 52 ms; acquisition voxel size  = 2×2×2 mm^3^; rec voxel size  = 1.75×1.75×2 mm isotropic, 60 axial adjacent slices; slice thickness  = 2 mm (no gap); FOV  = 224 mm×224 mm×120 mm; acquisition matrix  = 112×112; SENSE reduction factor  = 2, combined with a half-scan acquisition; 1 image without diffusion weighting and 15 diffusion-encoding gradients applied in 15 noncollinear directions; b-value  = 800 s/mm^2^; both the b0 and the 15 diffusion weighted images were averaged twice; bandwidth  = 2971 Hz/pixel; total acquisition time  = 7 min 34 sec.

A T2-weighted fluid attenuation inversion recovery (FLAIR) sequence was also acquired to ensure that vascular pathology was not significant. All images were rated using the Fazeka scale [48]. The mean and SD for all participants was 1.21, SD: 0.58;while specific subgroups were as follows: Controls: 1.18, SD 0.51; MCI:1.25, SD 0.67.

### High Resolution T1W Structural Image Processing

Images were skull stripped with the Brain Extraction Tool (BET) from the FSL library [49]. Brain tissue volume, normalized for subject head size, was estimated with SIENAX [50], [51], which is part of the FSL library. SIENAX starts by extracting brain and skull images from the single whole-head input data. The brain image is then affine-registered to MNI152 space [52], [53] (using the skull image to determine the registration scaling); this is primarily in order to obtain the volumetric scaling factor, to be used as a normalization for head size. The scaling factor is derived from the normalisation transformation matrix [51]. Next, tissue-type segmentation with partial volume estimation is carried out [54] in order to calculate total volume of brain tissue including separate estimates of volumes of WM and GM. For normalised and unnormalised GM and GM volumes analysis of variance (ANOVA) tests were carried out with GM or WM volume as the dependent variable and sex and diagnosis as the independent variables. If significant results were returned from these ANOVAs post-hoc Tukey tests were then performed to assess pair-wise differences between groups.

### DTI Processing

DTI analysis was performed using TBSS [55]. Images were skull stripped with the Brain Extraction Tool (BET) from the FSL library [49]. Raw DTI images were first corrected for motion and eddy current effects. The diffusion tensor was then calculated with the DTIFIT program for whole brain volumes and the resulting FA maps, together with the DA (λ1) and DR ((λ2+ λ3)/2) and MD ((λ1+ λ2+ λ3)/3) maps, were used in subsequent TBSS analysis.

TBSS performs a non-linear registration which aligns each FA image to every other one and calculates the amount of warping needed for the images to be aligned. The most representative image is determined as the one needing the least warping for all other images to align to it. The FSL library also provides a 1 mm isotropic FA target image (FMRIB58_FA) in standard space which sometimes used instead of the most representative image from the study cohort. This can be problematic as the target image is based on a young healthy brain. Using the method of “all subject to all subject” registration is more computationally intensively, but highly desirable when dealing with populations other than young healthy controls.

After this registration step, warped versions of each subject’s FA image were generated which were then averaged and a white matter “skeleton” is created suppressing all non-maximum FA values in each voxel’s local-perpendicular direction and subsequently comparing all remaining non-zero voxels with their nearest neighbours, thus searching for the centre of fibre bundles. The skeleton was then thresholded at an FA value of 0.2 which limits the effects of poor alignment across subjects and ensures that GM and CSF voxels are excluded from the skeleton. The skeleton that is now created contains WM tracts that are common to all subjects. A “distance map” is then created which is used to project each FA image onto the mean FA skeleton that is common to all subjects [55]. The same non-linear transformations derived for the FA maps were applied to the DA, DR and MD maps.

### Statistical Analysis

For statistical analysis, the images were analyzed using the “randomise” tool from FSL with a standard GLM design which controlled for the effects of age by including it as a covariate in the GLM design. The GLM also included additional explanatory variables which controlled for the differences between groups in terms of scores from the following tests: Word List Average, Word Recall and Praxis Recall. The GLM examined the main effect of sex and diagnosis, as well as a sex by diagnosis interaction. Pairwise differences between diagnostic groups, and also between subgroups (e.g. male controls versus male MCIs) were calculated. Randomise computes permutation tests on the assumption that the null hypothesis implies complete exchangeability of the observations [56]. Using this setup voxelwise differences between groups were then assessed, setting the number of permutations at 5000 permutations. Significance was tested at p<0.05 levels, corrected for multiple comparisons using the “2D” parameter settings with threshold-free cluster enhancement (TFCE), a method which avoids using an arbitrary threshold for the initial cluster-formation [56]. Boxplots of global diffusion with values extracted from the entire white matter skeleton were also created.

## Results

### Normalised Grey Matter and White Matter Volumes and Absolute Brain Volume

**Figure 1:**
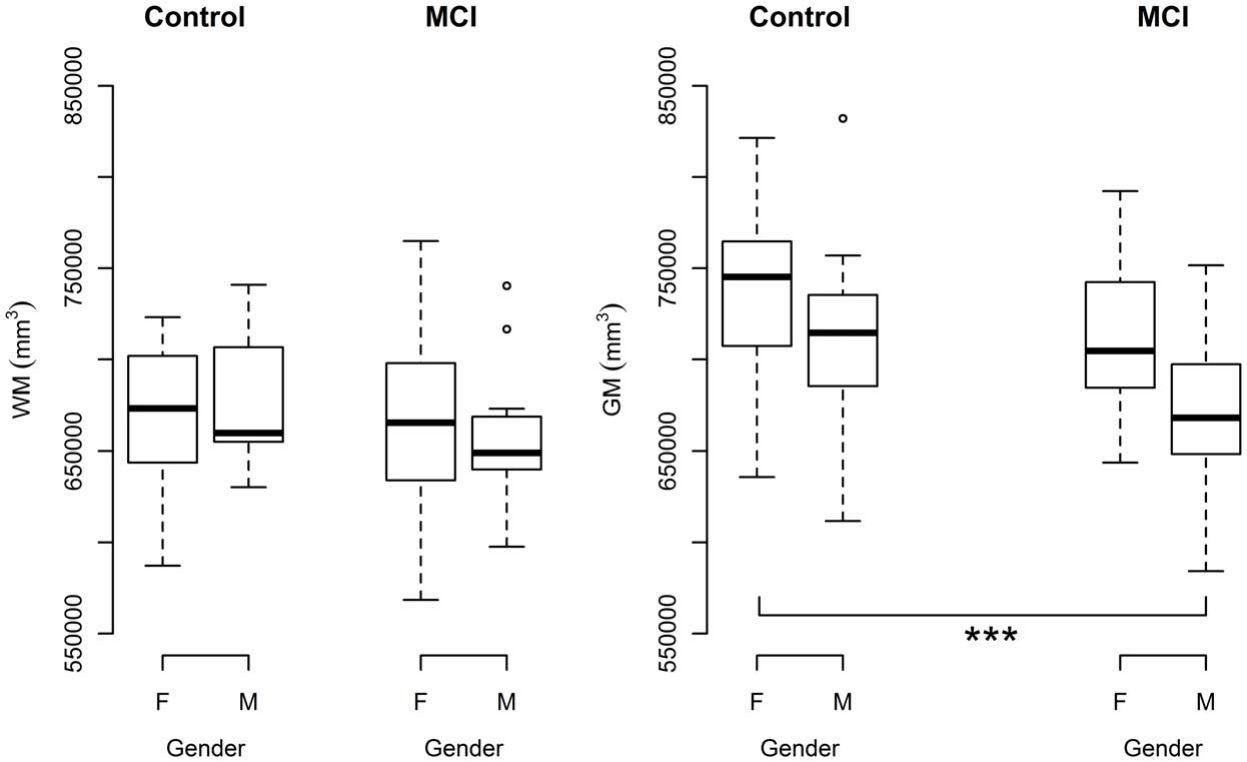
Sex differences for normalised grey matter volume and normalised white matter volume in control and MCI subjects. (A) No significant differences in white matter volume were found between males and females for control or MCI conditions. (B) Male MCI subjects had significantly lower GM volume relative to male controls. *** p<0.001, with post-hoc Tukey test, following an ANOVA.

An ANOVA test for normalised GM modelled against diagnosis by sex (i.e. normalised GM ∼ diagnosis × sex) returned a significant main effect of sex (F(1,68) = 7.86, p = 0.0066) and a significant main effect of diagnosis (F(1,68) = 7.87, p = 0.0066). No significant age by sex interaction was found. A post-hoc Tukey test found that normalised male MCI GM was significantly lower than normalised female control GM (p = 0.00085) (Fig. 1).

For an ANOVA test of normalised WM ∼ diagnosis × sex, no significant main effect of diagnosis or sex was found, and no significant interaction was found between these two factors (Fig. 1).

**Figure 2:**
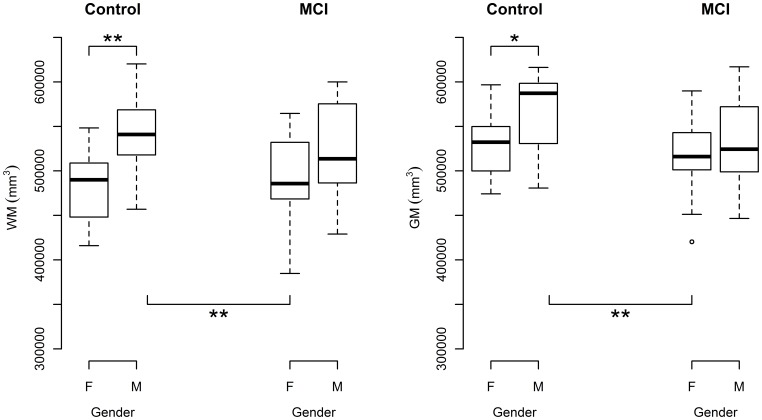
Sex differences for absolute grey and white matter volumes in control and MCI subjects. (A) Absolute male control WM volume is significantly larger than absolute female control white matter volume. Absolute male control WM volume is also significant larger than absolute female MCI WM volume. (B) Absolute male control GM volume is significantly larger than absolute female control grey matter volume. Absolute male control GM volume is also significant larger than absolute female MCI WM volume. *p<0.05, **p<0.01, *** p<0.001, with post-hoc Tukey test, following an ANOVA.

For absolute brain volume (i.e. non-normalised, gross brain volume), an ANOVA test returned a significant main effect of sex on absolute WM volume (F(1,68) = 18.88, p<0.0001) for the model WM volume ∼ diagnosis × sex. No significant main effect of diagnosis and no diagnosis by sex interaction was found. A post-hoc Tukey test, reported significant differences between male controls and female controls (p = 0.001), and also between male controls and female MCIs (p = 0.003) (Fig. 2).

For absolute GM volume, an ANOVA test returned a significant main effect of sex (F(1,68)  = 8.4958, p = 0.0048). The main effect of diagnosis on absolute GM volume was found to be borderline significant (F(1,68)  = 3.91, p = 0.052). There was no significant interaction between diagnosis and sex. A post-hoc Tukey test revealed a significant difference between male and female controls (p = 0.022). There was also a significant difference between male controls and female MCIs (p = 0.0034) (Fig. 2).

### Significant Differences between Healthy Older and MCI Participants in all Indices of Diffusion

**Figure 3:**
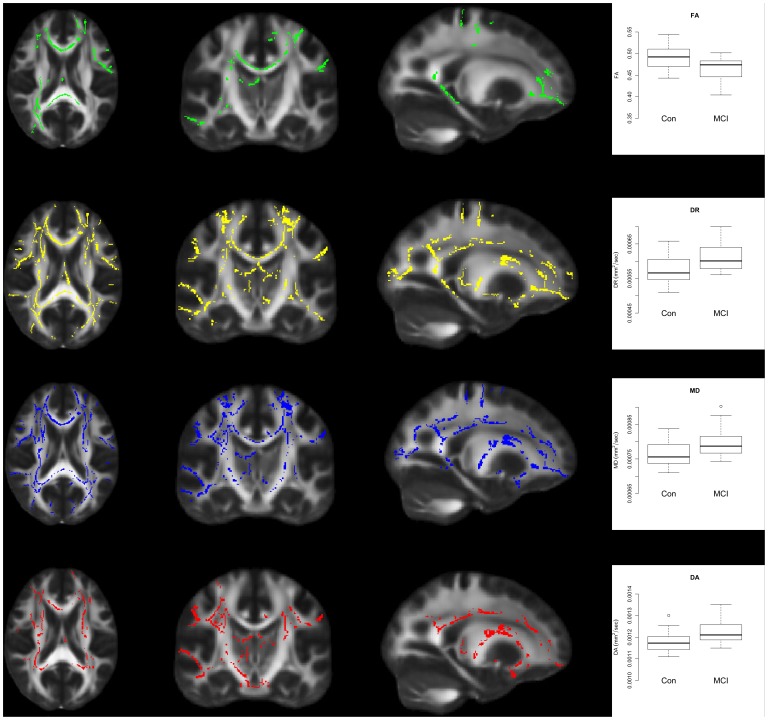
Differences in Multiple Indices of Diffusion between Control and MCI. FA (green) was significantly decreased in MCI subjects relative to controls, while DR (yellow), MD (blue) and DA (red) were significantly increased in MCI subjects relative to controls. The plots show values for each index of diffusion taken from the regions of the WM identified in the TBSS images. The TBSS images show results at p<0.05 corrected for multiple comparisons. The leftmost images show axial slices (z = 88), images in the central panel show coronal slices (y = 109) and the rightmost images show sagittal slices (x = 112).

Control participants were found to have significantly less FA values compared with MCI (Fig. 3). These significant decreases were found in the temporal lobe along the parahippocampal tract, the forceps major and forceps minor, and in the prefrontal cortex including the uncinate fasciculus.

For DA, DR and MD, these indices of absolute diffusion were all found to be significantly increased in MCI relative to controls (Fig. 3). Significant increases in these indices were more widespread than the regions of decreased FA noted above. Anatomical regions implicated include large portions of the temporal and parietal lobes, the prefrontal cortex, the superior and inferior longitudinal fasciculus. The forceps major and minor were implicated for DR and MD indices, but only the forceps minor for the DA index (Fig. 3).

### Significant Main Effect of Sex: FA Decreases, and MD and DR Increases in Men Relative to Women

**Figure 4:**
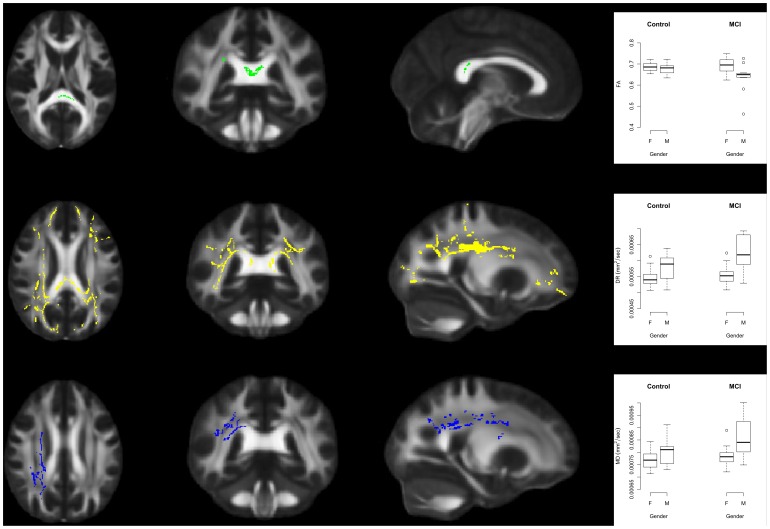
Significant main effect of sex. Top: Male subjects have lower FA than female subjects in the regions indicated in green. The plot shows the FA values in the significant TBSS regions highlighted in green. Middle: Male subjects have greater DR than female subjects in the regions indicated in yellow. The plot shows the DR values in the significant TBSS regions highlighted in yellow. Lower: Male subjects have greater MD values in the significant TBSS regions highlighted in blue. The plot shows the MD values in the significant TBSS regions highlighted in blue. No significant main effect of sex was found for the DA index. The TBSS images show results at p<0.05 corrected for multiple comparisons. For the FA images the axial slice is located at z = 88, the coronal slice is located at y = 89 and the sagittal slice is located at x = 93. For the DR images the axial slice is located at z = 97, the coronal slice is located at y = 86 and the sagittal slice is located at x = 65. For the MD images the axial slice is located at z = 101, the coronal slice is located at y = 84 and the sagittal slice is located at x = 66. Abbreviations in the boxplots: M: male; F: female.

The general linear model applied to TBSS results indicated a significant main effect of sex for FA, DR and MD, with males having significantly decreased FA and significantly increased DR and MD relative to women in the anatomical regions indicated in Figure 4. No significant main effect of sex was found for the DA index.

As can be seen in Figure 4, the regions of decreased FA in male participants relative to female participants lie predominantly within the splenium for the FA index. Areas implicated for the DR index include the SLF and ILF bilaterally, the splenium, anterior thalamic radiation, forceps minor and uncinate fasciculus bilaterally. Areas implicated for the MD index include the right superior longitudinal fasciculus (SLF) and the right inferior longitudinal fasciculus (ILF).

### Significant Main Effect of Age: FA Decreases and DR, MD, and DA Decreases with Age

**Figure 5:**
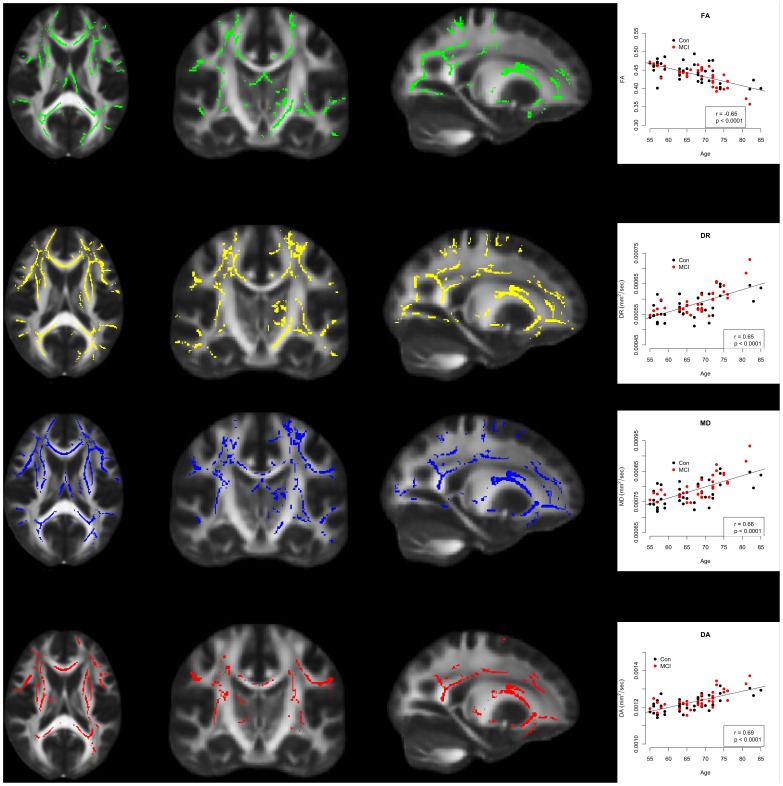
Significant effect of age on all indices of diffusion. FA (green) was found to decrease significantly with age. The plot of FA versus age utilises FA values taken from the green regions highlighted. DR (yellow) was found to increase significantly with age. The plot of DR versus age utilises DR values taken from the yellow regions highlighted. MD (blue) was found to increase significantly with age. The plot of MD versus age utilises MD values taken from the blue regions highlighted. DA (red) was found to increase significantly with age. The plot of DA versus age utilises DA values taken from the yellow regions highlighted. The leftmost images show axial slices (z = 88), images in the central panel show coronal slices (y = 109) and the rightmost images show sagittal slices (x = 112).

The GLM returned a significant main effect of age. FA values decreased significantly and DR, MD and DA values decreased significantly in the regions indicated in Figure 5. The anatomical regions implicated were widespread including sections of the parietal, occipital and temporal lobes, as well as significant portions of the prefrontal cortex and the uncinate fasciculus (Fig. 5). There was no significant age by sex interaction.

### Regression Analysis for Age versus Normalised White Matter Volume and Grey Matter Volume

**Figure 6:**
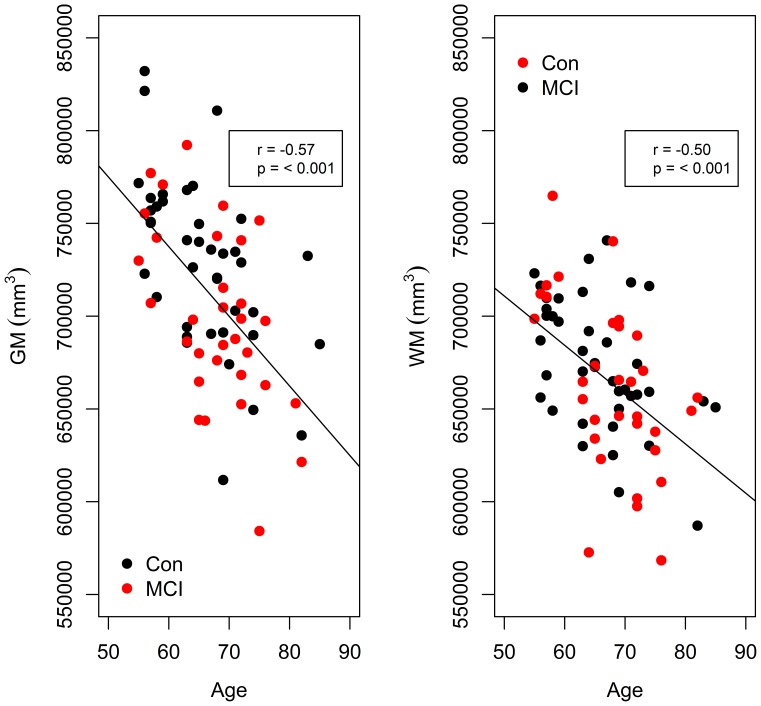
Grey matter and white matter volume decline with age. An ANOVA model with normalised GM volume as the dependent variable and age, sex and diagnosis as the independent variables, returned a significant main effect of age, and no significant interactions. Here, a regression line for GM decline with age (including men and women) is plotted (left). An ANOVA model with normalised WM volume as the dependent variable and age, sex and diagnosis as the independent variables, also returned a significant main effect of age, with no significant interactions. A regression line for WM decline with age (including men and women) is plotted (right). The r value denotes the correlation as calculated by Pearson’s product-moment correlation using data from all subjects. Correlations are significant as indicated by the p values.

An ANOVA test for normalised GM volume ∼ age × sex × diagnosis, returned a significant main effect of age (F(1,64)  = 34.16, p<0.00001), and a significant main effect of diagnosis (F(1,64)  = 5.89, p = 0.018). No significant interactions were returned. A regression analysis for normalised GM volume decreases with age is shown in Figure 6 (r = −0.57, p<0.001).

An ANOVA test for normalised WM volume ∼ age × sex × diagnosis returned a significant main effect of age (F(1,64)  = 24.25, p<0.00001). No significant interactions were returned. Similar to normalised GM volume, normalised WM volume decreases significantly with age (r = −0.50, p<0.001) (Fig. 6).

## Discussion

To the best of our knowledge, this is the first study to examine sex differences in those with MCI in terms of DTI measures of WM structural integrity. Our initial findings of significantly lower FA, and significantly higher DR, MD and DA in MCI participants relative to healthy older controls are broadly consistent with previous DTI studies [17], [23]–[25], [57], [58]. However, we also report a significant main effect of sex which has not been previously noted. This significant main effect of sex demonstrated that men have significantly raised DR and MD, and significantly lower FA, relative to women, both within healthy older and MCI participants. There was no sex by diagnosis interaction, indicating that increased WM microstructural damage in men relative to women at an equivalent cognitive status is constant within both healthy older and MCI participants. The main effect of sex was found to have a degree of laterality, with the right hemisphere being more affected than the left.

There have been extremely few studies investigating WM differences between men and women in older populations, but those that exist have reported a limited degree of sexual dimorphism [59], [60]. The differences between the current findings and those of Wu and colleagues, and Hsu and colleagues, may lie in the fact that these previous works report cross-sectional decline in WM microstructural parameters in men and women over the life span, whereas the current study focuses on sex differences within an age-matched elderly cohort. Sex differences in multiple indices of diffusion have not been systematically studied. Some DTI work in younger subjects showed no sex differences [61], [62] whereas others did show significant sex differences in young subjects though only when focusing on predefined brain regions, such as frontal lobe or corpus callosum [36], [63], [64]. However, studies involving young subjects do not relate well to our older cohort.

Previous PET studies in AD dementia patients have noted that men have a pronounced hypermetabolism relative to women, particularly in the right hemisphere [11], [65]. These findings are relevant to the current results which indicate that the degree of WM damage is greater in the right hemisphere of male participants. Interestingly, an early PET study also noted that men tend to have greater metabolism in the right hemisphere than the left after the age of 60 [66].

While the underlying reasons for this laterality are not known it is of interest that a model of aging, termed HAROLD (hemisphere asymmetry reductions in older adults) posits that there is an age-related loss of hemispheric asymmetry of task-related activation in the prefrontal cortex (PFC). While healthy young subjects show greater activation in left PFC compared with the right PFC during the execution of a variety of cognitive tasks, many studies indicate that this laterality is reduced in older subjects [67]–[71]. This loss of laterality is frequently related to increased right PFC activation in older adults which has been suggested to serve as a possible compensatory mechanism in order to maintain cognitive performance in older age. The large number of functional activation studies showing increased right hemisphere functional activation in older age [67]–[71] may serve as indirect evidence for chronically stressed right hemisphere structures being predilection sites for structural alterations in the early stages of neurodegeneration. A number of recent MCI and AD dementia classification studies have also noted a right-more-than-left pattern of WM damage [3], [72], [73]. It is possible that while men are “chronologically” age-matched with women, the fact that women live longer than men on average [74] could potentially result in men being “biologically” older than women of the same age which may account for a degree of extra WM damage in the right hemisphere of older men relative to women.

**Figure 7:**
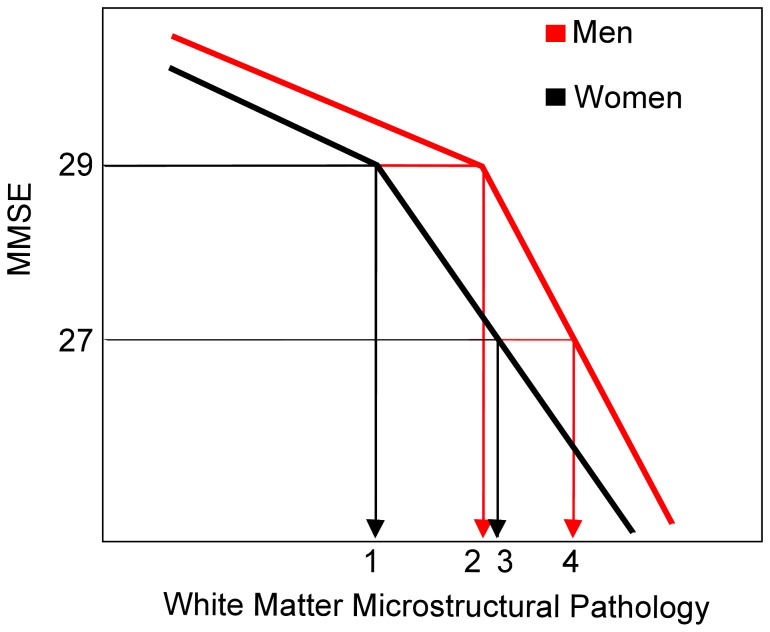
Model of sex-specific white matter degeneration. Healthy older subjects (male and female) have an MMSE of ∼29. However, at an equivalent MMSE score, healthy older women (1) have less microstructural damage than healthy older men (2). As we enter the MCI spectrum, female MCIs (3) again have less microstructural damage than male MCIs (4). It is worth noting that the level of microstructural damage of female MCI subjects in the current study was equivalent to the level of damage found in male control subjects.

Another possible explanation for the overall greater structural damage in men may be that brain reserve is greater in older men compared with older women. Although men have more structural damage than women, they are at the same cognitive level as women, suggesting that women may manifest the symptoms of cognitive decline at a level of structural damage which would not cause the same amount of cognitive decline in men. This can be seen in the scheme proposed in Figure 7 where healthy older men have the equivalent microstructural WM status as women with MCI.

Brain reserve refers to the fact that larger brains can sustain more structural damage before clinical deficits emerge because sufficient neural substrate for normal function is retained in larger brains following an insult [4], [13]. It has been proposed that when brain reserve is depleted beyond a critical threshold, then specific clinical or functional deficits may emerge [13]. In the current study, this critical threshold appears to be higher in older men compared with older women. The fact that men have a greater absolute brain size, as well as a higher density of neurons compared with women may contribute to this proposed extra brain reserve in men [7], [8].

In the current study, DA was found to increase in MCI, a finding which is at odds with animal studies and human callosotomy studies which have shown that axonal pathology results in a decrease in axial diffusion [75]–[77]. Yet raised DA in MCI and AD dementia has consistently been found in recent DTI studies [17], [24], [25], [58], [78]. The complexity of DA responses may reflect the fact that WM damage has been shown to lead to an initial decrease in DA followed by subsequent increases as axonal fragments are cleared by microglia and water molecules can diffuse longitudinally again [79], [80]. Also, where two crossing fibre tracts exist, it may be difficult to dissect out the axial diffusion of one of the two fibre tracts. Thus, increases in DA may reflect a degeneration of one of the two fibre populations in regions of crossing fibers [81].

WM and GM volumes were also found to have sex specific profiles. Male MCI participants showed significantly lower normalised GM volume than female MCI participants. Again, this suggests that for an equivalent diagnosis of MCI, men must sustain more GM atrophy than women in order to be diagnosed as MCI. Therefore, the threshold of structural damage which precipitates cognitive decline leading to a diagnosis of MCI appears to be lower in women. No sex-specific differences were found in normalised WM volume which suggests that while there are significant WM microstructural changes in male MCI participants, this has not reached the point of gross tissue loss. This result also highlights the fact that DTI has the ability to detect WM microstructural changes which may develop before global WM changes occur. This is particularly true in MCI, where the structural changes are subtle. In an earlier study we also noted diffusion changes in healthy older, MCIna and MCIa subjects, in the absence of significant changes in normalised WM volume [19]. However, in the situation of AD dementia both WM volume and diffusion indices were significantly affected [19].

Absolute WM and GM volume were both greater in male controls relative to female controls, which is consistent with previous studies [5], [6]. No significant differences were found however, between absolute volumes of male and female MCI participants, or between absolute male MCI volumes and male controls, or between absolute female MCI volumes and female controls. These results concur with the hypothesis that the greater number of neurons in male brains may create a buffer against pathology translating into clinical symptoms of AD [7], [8]. This possibility of greater brain reserve in males may help to delay the onset of clinical symptoms of dementia in males [1], [9]–[11], [13]. Thus healthy older men would have to reach a higher threshold of damage before expressing the earliest symptoms of neurodegeneration.

Although there have been conflicting results, the majority of studies also indicate that the incidence of AD dementia is greater in women than in men [82]–[86]. At least one large meta-analysis confirmed that the risk of AD dementia is increased 1.6 fold in women [82]. While caution is needed when relating the results of AD dementia studies to MCI, it is nevertheless reasonable to suggest that these studies are indicative of a greater vulnerability of women to neurodegeneration in older age. With 10–15% of MCI subjects converting to AD dementia, our finding that women may be more vulnerable than men to transition to MCI, may in turn lead to more women deteriorating to AD dementia than men.

Genetic and hormonal characteristics might contribute to the extra vulnerability of older women. Oestrogen has been shown to have a potent neuroprotective effect, and it is possible that there is a profile of increased protection from neurodegeneration while circulating oestrogen levels are normal, followed by increased risk of neurodegeneration in older women as oestrogen levels decline following menopause [86], [87].

In addition to brain reserve, the role of cognitive reserve should also be noted. While brain reserve is a passive model that highlights the role of brain size or neuronal volume, cognitive reserve is an active model which suggests that the brain responds to brain damage by recruiting existing cognitive processes or by enlisting compensatory processes [88], [89]. In a situation where two people have the same brain reserve capacity, the person with more cognitive reserve may tolerate more neural damage before cognitive impairment is apparent. This model focuses on the processes that allow people to sustain brain damage and maintain function. Education and socioeconomic status such as income and occupational attainment have been shown to influence cognitive reserve [89], [90]. As the mean age of the current cohort is ∼70, our participants were of school-going age during the 1940’s and were potentially in employment from the late 1950’s onwards. Significant differences in gender roles and opportunities may have disadvantaged women at this time. Although there were no significant differences between men and women in terms of years of education, both the quantity and quality of jobs available to women are likely to have been more limited for women. Also, aspects of occupational experiences have been found to impart additional cognitive reserve over and above that obtained from education [13]. Thus, it is possible that within the current cohort, social and cultural factors may have negatively impacted the opportunities for women and led to lower occupational attainment, which in turn may negatively affect cognitive reserve. We should note that models of cognitive reserve and brain reserve are not mutually exclusive and may be interdependent [89].

Strikingly, Barnes and colleagues recently noted, using data from the Religious Orders Study, that the association between AD pathology and clinical AD was significantly stronger in women than in men [3]. It was also noted that each unit of AD pathology increased the odds of clinical AD more than 20-fold in women compared with a three-fold increase in men. The authors suggested that AD pathology is more likely to be clinically expressed as dementia in women than in men. This relates to the findings in the current study, although caution is needed when applying the results of AD studies to the current MCI findings, particularly as we do not have follow-up data and thus do not know which MCI participants remained stable and which MCI participants progressed to AD dementia.

In terms of the rate of deterioration of WM microstructure with age (as measured by FA, DA, MD and DR), no age by sex interaction was found. The areas implicated in microstructural deterioration with age were dispersed through the brain, with a slightly more pronounced effect in frontal regions which is consistent with the concept of an anterior-posterior gradient of WM deterioration with age [91], [92]. The lack of any age by sex interaction for microstructural deterioration with age has been consistently noted by other studies [59], [93], [94].

Similarly, the rate of decline of GM with age was not found to be significantly influenced by sex which is also consistent with earlier work that has shown similar rates of GM volume decline in men and women which agrees [95], [96]. Previous studies investigating the effect of sex on the rate of WM volume decline with age in elderly populations are scarce and results have been equivocal [27], [97]–[99] but some evidence suggests that men show greater tendency for age-related differences in WM volume than women [100], [101]. The WM shrinkage with age identified in the current study is consistent with age-related changes reported in MRI relaxation times [98].

A limitation of the current study is the relatively small number of participants included. A study with a larger sample size of MCI participants is warranted to verify and further probe the impact of sex on mild cognitive impairment. This is being pursued as part of the European DTI Study in Dementia (EDSD) initiative. The small size of the current sample may weaken the ability to reliably identify interactions between sex and age, as well as, interactions between sex and diagnosis. It is now planned to assess these interactions in an independent sample with larger numbers of participants.

The cross-sectional nature of this study should be noted. We do not have follow-up data and thus do not know which participants subsequently developed AD or alternatively remained stable without deteriorating further. Therefore, the heterogeneity of the current cohort and the lack of diagnostic certainty in the cohort should be borne in mind. Future studies should also consider sex differences between those with MCI that remain stable and those that deteriorate to AD dementia. Technical limitation of the methodology should also be noted. Although TBSS strives to avoid the problems of voxel based morphology relating to partial voluming, some of these issues may still remain. Small WM tracts may be contaminated with GM if the tract width is smaller than the original voxel size, although applying a threshold of 0.2 or less to the WM skeleton in the TBSS preprocessing steps is thought to remove the potential occurrence of GM.

Overall our results show that in both control and MCI groups, men have greater WM damage than women with the same clinical diagnosis. This suggests that the threshold of WM damage that must be reached before entering the MCI spectrum is lower in women compared with men. The identification of older people with mild cognitive impairment that are at high risk of evolving to AD is vitally important for early treatment. The concept of MCI is now widely used to describe this high-risk group, and numerous research programmes have been undertaken with the aim of providing therapeutic intervention at the MCI stage which might reduce or prevent the possibility of progressing on to AD dementia. The current results indicate that sex differences need to be carefully considered when developing therapeutic strategies for the treatment of AD. While sex may have been overlooked in the past by simply including sex matched groups, the current study points to a need to take sex into account in all drug trials which seeks to target AD dementia and reduce its incidence in the population.
